# Characterization of pathogenic bacterial distribution in extracorporeal membrane oxygenation-related nosocomial infections and the prognostic value of early inflammatory biomarkers for infection survival

**DOI:** 10.3389/fmicb.2025.1555701

**Published:** 2025-03-04

**Authors:** Zhiwen Zhao, Pengfei Liang, Lanlan Cai, Li Zhang, Qi Jia, Wentao Tao, Zhicheng Fang

**Affiliations:** Department of Emergency Medicine, Taihe Hospital, Hubei University of Medicine, Shiyan, China

**Keywords:** extracorporeal membrane pulmonary oxygenation, nosocomial infection, inflammatory markers, prognosis, rescue

## Abstract

**Introduction:**

Extracorporeal membrane pulmonary oxygenation (ECMO) is the last barrier to save lives and is widely used in the treatment of critical respiratory and circulatory diseases, but infection is one of its common complications. The aim of this study was to analyse the clinical characteristics, survival rates and prognostic factors of patients with ECMO-related nosocomial infections.

**Methods:**

This study retrospectively analysed patients treated with ECMO at a tertiary hospital in China between 2017 and 2023. Patient demographic data, ECMO indications, type of pathogen and site of infection, duration of ECMO and tracheal intubation-assisted breathing, and indicators of inflammation at the time of first infection were collected. Patients were divided into surviving and non-surviving groups based on survival, and differences in early inflammatory markers between the two groups were compared.

**Results:**

A total of 186 patients were treated with ECMO between 2017 and 2023, of whom 61 (32.7%) developed nosocomial infections and 5 declined to participate in the study. In the surviving group after infection, 21 patients (37.5%) had a mean age of 51 years; in the non-surviving group, 35 patients (62.5%) had a mean age of 54 years. The most common site of infection was the respiratory tract (75%), followed by haematogenous infections; the predominant pathogenic organisms were *Acinetobacter baumannii* (46.43%) and *Klebsiella pneumoniae* (35.71%). IL-6, hs-CRP, and Plt differed significantly between the two groups (*p* < 0.05) [IL-6 (40.62 vs. 196.75 μg/mL, *p* < 0.001), hs-CRP (8.86 vs. 23.60 mg/L, p < 0.001), and Plt (85.00 vs. 48.50 × 10^9^, *p* = 0.02)], but there were no significant differences in PCT, WBC, and NE. One-way logistic regression analysis showed that IL-6 (OR: 1.02, 95% CI: 1.01–1.03; *p* = 0.001), hs-CRP (OR: 1.59, 95% CI: 1.02–2.47; *p* = 0.041), and Plt (OR: 1.01, 95% CI: 1.01–1.02; *p* = 0.031) were important factors affecting the prognosis of ECMO-related nosocomial infections.

**Conclusion:**

Respiratory tract infections were the most common during ECMO treatment, and the main pathogen was *Acinetobacter baumannii*. Early inflammatory markers such as elevated IL-6, hs-CRP and reduced platelet count may be risk factors for poor prognosis and have significance in guiding prognostic assessment.

## 1 Introduction

Extracorporeal membrane pulmonary oxygenation (ECMO) can provide continuous and stable cardiopulmonary function in acutely ill patients when traditional means of salvage fail, offering a glimmer of hope for overcoming the disease (Perman et al., 2024; Zhao and Fang, 2024). The frequency of ECMO-assisted life-saving treatment has increased significantly over the past decade, especially during the COVID-19 pandemic in 2019 (Tonna et al., 2024). Although clinicians have gained some experience with ECMO, it is not without risk (Kim et al., 2021), and its complications are significantly associated with prognosis (Cheng et al., 2024); among them, hospital-acquired infections are one of the most common and serious complications of ECMO (Tavares et al., 2023); extracorporeal membrane pulmonary oxygenation-acquired hospital-acquired infections have been reported in up to 64% of adult and 30% of neonatal ECMO cases (Selçuk et al., 2021; Bizzarro et al., 2011). Ventilator-associated pneumonia (VAP) and bloodstream infections (BSIs) were the most frequent sources of hospital-acquired infections in patients on ECMO, accounting for 33% and 15%, respectively (Ait Hssain et al., 2024).

Previous studies have shown that risk factors associated with increased ECMO infections include (Kim et al., 2021): duration of continuous ECMO support, severity of underlying disease, and prolonged intensive care unit stay. However, symptoms of infection in ECMO patients are often non-specific and easily masked by extracorporeal membrane pulmonary oxygenation itself, thus making the diagnosis of infection challenging (Abrams et al., 2020). Meanwhile, there is no unified understanding of ECMO-related nosocomial infections, from clinical diagnosis and treatment to prevention (Bizzarro et al., 2011).

Recent research have shown that elevated creatinine levels during ECMO support are associated with an increased risk of infection. Research has identified higher baseline creatinine values as an independent risk factor for death 4 h after ECMO initiation (Mornese Pinna et al., 2023). Additionally, elevated lactate levels have been found to increase mortality in patients with ECMO-related nosocomial infections (Sun et al., 2017). Interestingly, while body mass index (BMI) in patients on ECMO was not associated with the risk of bloodstream infections, patients with a higher BMI had lower in-hospital mortality associated with ECMO support (Lee et al., 2023). For adult patients receiving VA-ECMO after cardiac surgery, nomogram-based monitoring tools can be used in clinical practice to identify those at risk of hospital-acquired infections and provide early warnings (Li et al., 2024). Although the aforementioned studies have contributed to the diagnosis and management of ECMO-related nosocomial infections, the predictive value of early inflammatory markers for prognostic assessment during ECMO therapy remains underexplored.

The present study was a retrospective analysis aimed at elucidating the clinical epidemiological characteristics of the surviving and non-surviving groups of patients who received ECMO for the first time in adults and developed nosocomial infections. By analysing early inflammatory markers, we hope to provide a basis for predicting patient prognosis and developing interventions to reduce mortality in patients with ECMO-related nosocomial infections.

## 2 Research methods

### 2.1 Study design and population

This study was a retrospective analysis, and the study site was Taihe Hospital Affiliated to Hubei University of Medicine. The study population consisted of 186 patients who received ECMO-assisted therapy between January 2017 and December 2023 in this hospital. Forty-five of them survived, with a survival rate of 24.1%. Sixty-one of these patients (32.7%) developed ECMO-complicated nosocomial infections, and five patients declined to participate in the study, resulting in the inclusion of 56 patients. ECMO mode (VV-ECMO or VA-ECMO) was selected based on the patient’s condition. A total of 55 patients underwent cannulation via the right femoral artery and femoral vein, while one patient underwent cannulation via the femoral artery and jugular vein. VV-ECMO was primarily used in patients with refractory hypoxemia, hypercapnia, and ARDS after aggressive treatment, while VA-ECMO was mainly applied in patients with severe cardiogenic shock, cardiac arrest, viral or fulminant myocarditis, severe arrhythmia, cardiac tachyarrhythmia, severe cardiomyopathy, and heart failure.

We recorded patient demographics, ECMO indications, source of infection and pathogenic microorganism characteristics, time to first infection, ECMO run time, time to tracheal intubation, stage of infection, invasive procedures, and inflammatory markers at the time of the first infection, including interleukin 6 (IL-6), procalcitonin (PCT), high-sensitivity C-reactive protein (hs-CRP), white blood cell count (WBC), and neutrophil count (NBC), neutrophil percentage (NE%) and platelets (Plt). The study was approved by the Medical Ethics Committee of Taihe Hospital Affiliated to Hubei University of Medicine (Ethical Review No. 2024KS33) and was conducted in accordance with the Declaration of Helsinki.

### 2.2 Relevant definitions, inclusion criteria, and exclusion criteria

#### 2.2.1 Definition of ECMO-associated nosocomial infections and overall survival rate

The definition of ECMO-associated nosocomial infections was based on the Hospital Infection Diagnostic Criteria (Recommendations) issued by the Ministry of Health of the People’s Republic of China in 2001 (which refers to the definition of the Centers for Disease Control and Prevention (CDC) 1988 NI) (Li et al., 2023; Garner et al., 1988). Nosocomial infections during ECMO were defined as infections occurring during the period of 24 h after the activation of the ECMO to 48 h after the removal of the ECMO (Li et al., 2023; Wang et al., 2021; Schmidt et al., 2012). Bloodstream infections were determined primarily on the basis of blood cultures, with additional blood anaerobic or fungal cultures for suspected blood anaerobic or fungal infections (Zingg et al., 2019; Yangco, 1989). Infections of respiratory origin are identified primarily on the basis of culture from bronchoalveolar lavage or endotracheal aspirates (Spalding et al., 2017). Urinary tract infections were defined as at least one positive urine culture (>10^5^ microorganisms/mL) (Wagenlehner et al., 2024). In addition, we included PCR diagnosis, and mNGS pathogenetic diagnosis (Han et al., 2019).

#### 2.2.2 Inclusion and exclusion criteria for ECMO-associated nosocomial infections

##### 2.2.2.1 Inclusion criteria

(1) Age ≥ 18 years; (2) Patients who received ECMO-assisted therapy between January 2017 and December 2023; (3) ECMO-assisted therapy time ≥ 48 h; (4) Diagnosis of infection was confirmed by pathogenic bacterial culture, PCR diagnosis, and mNGS pathogenicity diagnosis; (5) No signs of infection before ECMO-assisted therapy are ruled out based primarily on the patient’s history, clinical presentation, laboratory tests (e.g., blood cultures, C-reactive protein, white blood cell count), and imaging tests (e.g., chest X-ray or CT scans).

##### 2.2.2.2 Exclusion criteria

(1) Infection existed before ECMO treatment; (2) patients who received ECMO treatment in other hospitals; (3) patients and their families refused to participate; (4) decannulation or death within 48 h of ECMO support treatment.

### 2.3 Overview of ECMO management protocol and infection prevention measures

The ECMO management protocol follows the Extracorporeal Life Support Organization (ELSO) guidelines (Conrad et al., 2018; Broman et al., 2019; Lorusso et al., 2021). Successfully discharged from ECMO-assisted supportive therapy and survived for more than 48 h (Fernando et al., 2023). All patients included in this study received ECMO-assisted therapy, tracheal intubation, mechanical ventilation, central venous catheterization, gastric tube insertion, and urinary catheter placement. Regarding antibiotic use, bacterial cultures and infection prophylaxis were as follows: First-generation cephalosporins were administered prophylactically prior to ECMO cannulation and continued for 24 to 48 h postoperatively. In clinically stable patients without signs of infection, treatment was discontinued after less than 24 h based on clinical judgment. Piperacillin-tazobactam therapy was initiated when infection was suspected, specifically in cases presenting with persistent high fever, markedly elevated inflammatory markers, early blood culture or bronchoalveolar lavage fluid (BALF) culture results indicating a first-generation cephalosporin-resistant pathogen, abnormal imaging findings, or sepsis. No antifungal or antiviral prophylaxis was given to any of the patients. After ECMO initiation, daily samples were taken from the arterial and venous ends of the ECMO circuit, the respiratory tract, and the urinary catheter. Cultures, PCR diagnostics, and mNGS were performed for pathogen detection. Infection prevention measures included aseptic handling of all invasive procedures in hospitalized patients, daily observation of catheter positions, and regular replacement of materials used for invasive procedures.

### 2.4 Statistical analysis of data

Non-normally distributed measures were expressed as medians (interquartile spacing), and the Mann–Whitney U test was used to compare the medians of continuous variables between the two groups. Comparisons of categorical variables were performed using the *χ*^2^ or Fisher exact test, and one-way logistic regression was used to analyse early inflammatory markers in patients with ECMO-associated nosocomial infections. All tests of significance were two-sided, and differences were considered statistically significant with a *p* value <0.05.

## 3 Results

### 3.1 Demographic and clinical characteristics of patients with extracorporeal membrane oxygenation-associated nosocomial infections

A total of 56 patients with ECMO-associated nosocomial infections were included in this study (see Table 1). There were 21 patients in the survivor group with a median age of 51 years (26 to 56 years) and a median length of stay of 28 days (19 to 41 days), of whom 14 (66.6%) were male. In the non-surviving group, there were 35 people with a median age of 54 years (47 to 58 years) and a median length of stay of 10 days (3.5 to 19.5 days), 31 of whom were male (88.57%). Significant differences between the two groups were observed in total length of hospital stay, history of antibiotic use in the last month, and history of glucocorticoid and immunosuppressant use in the last month (all *p* < 0.05). In contrast, no significant differences were found between the groups regarding smoking and drinking history, history of operative procedures (including percutaneous coronary intervention, appendectomy, cesarean section, cholecystectomy, and spinal surgery), or underlying diseases (e.g., coronary heart disease, hypertension, chronic obstructive pulmonary disease, diabetes mellitus, chronic gastritis, chronic renal insufficiency, and cerebral infarction).

**Table 1:** Demographic and clinical characteristics of patients with hospital-associated infections in ECMO.

Characteristics	Survivor group (*n* = 21)	Non-survivor group (*n* = 35)	*p*
Gender, *n* (%)			0.099
Male	14 (66.67)	31 (88.57)	
Female	7 (33.33)	4 (11.43)	
Age (years)	51 (26, 56)	54 (47, 58)	0.106
Total length of hospital stay (days)	28 (19, 41)	10 (4.5, 19.5)	<0.001
History of smoking and alcohol	6 (28.57)	5 (14.29)	0.339
History of antibiotics in the last month	2 (9.52)	17 (48.57)	0.003
History of glucocorticoids and immunosuppressants in the last month	4 (19.05)	18 (51.43)	0.016
History of operative procedure, *n* (%)	12 (57.14)	21 (60.00)	0.833
Percutaneous coronary intervention	5 (23.81)	7 (20.00)	
Appendectomy	2 (9.52)	3 (8.57)	
Cesarean section	3 (14.29)	4 (11.43)	
Cholecystectomy	1 (4.76)	2 (5.71)	
Spinal surgery	1 (4.76)	3 (8.57)	
Other	0 (0.00)	2 (5.71)	
Underlying disease, *n* (%)			
Coronary heart disease	8 (38.10)	16 (45.71)	0.577
Hypertension	6 (28.57)	12 (34.29)	0.658
Chronic obstructive pulmonary disease	4 (19.05)	9 (25.71)	0.806
Diabetes	1 (4.76)	7 (20.00)	0.237
Chronic gastritis	4 (19.05)	1 (2.86)	0.116
Chronic renal insufficiency	2 (9.52)	1 (2.86)	0.646

### 3.2 Microbiological characteristics of etiology, ECMO modes, and ECMO-associated nosocomial infections in patients receiving ECMO therapy

Table 2 shows that the main diseases treated with ECMO included cardiac arrest in 18 cases (32.14%), ARDS in 16 cases (28.57%), septic shock in 7 cases (12.5%), traumatic shock in 6 cases (10.71%), fulminant myocarditis in 6 cases (10.71%), heart valve disease in 5 cases (8.93%), and aortic coarctation in 3 cases (5.36%). As shown in Table 3, VA-ECMO modes were primarily used for patients with Cardiac Arrest, Septic Shock, Traumatic Shock, Fulminant Myocarditis, Heart Valve Disease, Aortic Coarctation, and a small number of ARDS cases. VV-ECMO modes are predominantly used for ARDS patients. Table 4 shows that the most common site of infection for ECMO-associated infections was the respiratory tract (42 cases, 75%), followed by bloodstream infections (31 cases, 55.36%). Among the pathogenic microorganisms, Gram-negative bacteria accounted for the majority, including *Acinetobacter baumannii* in 26 cases (46.43%), followed by *Klebsiella pneumoniae* in 20 cases (35.71%). Among the gram-positive bacteria, Staphylococcus spp. were the most common with 14 cases (25%), including 7 cases (12.5%) of *Staphylococcus aureus*. There was no significant difference in the number of pathogenic microorganisms co-infected in the two groups of patients (*p* > 0.05).

**Table 2:** Etiologies receiving treatment with ECMO-assisted therapy.

Characteristics	Survivor group (*n* = 21)	Non-survivor group (*n* = 35)	*p*
Cardiac arrest, *n* (%)	8 (38.10)	10 (28.57)	0.46
Acute respiratory distress syndrome, *n* (%)	7 (33.33)	9 (25.71)	0.541
Septic shock, *n* (%)	1 (4.76)	6 (17.14)	0.348
Traumatic shock, *n* (%)	2 (9.52)	4 (11.43)	
Fulminant myocarditis, *n* (%)	2 (9.52)	4 (11.43)	
Heart valve disease, *n* (%)	1 (4.76)	4 (11.43)	0.717
Aortic coarctation, *n* (%)	1 (4.76)	2 (5.71)	
Other, *n* (%)	2 (9.52)	1 (2.86)	0.646

**Table 3:** Different ECMO modalities for patients with various etiologies.

Characteristics	Totals	VA-ECMO modes	VV-ECMO modes
Cardiac arrest, *n* (%)	18	18 (100.0)	
Acute respiratory distress syndrome, *n* (%)	16	2 (12.5)	14 (87.5)
Septic shock, *n* (%)	7	7 (100.0)	
Traumatic shock, *n* (%)	6	6 (100.0)	
Fulminant myocarditis, *n* (%)	6	6 (100.0)	
Heart valve disease, *n* (%)	5	5 (100.0)	
Aortic coarctation, *n* (%)	3	3 (100.0)	
Other, *n* (%)	3	2 (66.7)	1 (33.3)

**Table 4:** Microbiological characterization of sources of ECMO-associated nosocomial infections.

Characteristics	Survivor group (*n* = 21)	Non-survivor group (*n* = 35)	*p*
Source of pathogenic microorganisms, *n* (%)			
Respiratory tract	16 (76.19)	26 (74.29)	0.873
Blood	11 (52.38)	20 (57.14)	0.729
Urinary system	2 (9.52)	6 (17.14)	0.693
Gram-negative bacteria, *n* (%)			
*Acinetobacter baumannii*	11 (52.38)	15 (42.86)	0.489
*Klebsiella pneumoniae*	5 (23.81)	15 (42.86)	0.15
*Stenotrophomonas maltophilia*	1 (4.76)	4 (11.43)	0.717
*Pseudomonas aeruginosa*	3 (14.29)	1 (2.86)	0.284
*Haemophilus parainfluenzae*	1 (4.76)	0 (0.00)	0.375
Gram-positive bacteria, *n* (%)			
*Staphylococcus spp*	7 (33.33)	7 (20.00)	0.265
*Staphylococcus aureus*	3 (14.29)	4 (11.43)	
*Staphylococcus epidermidis*	2 (9.52)	1 (2.86)	
*Staphylococcus haemolyticus*	2 (9.52)	2 (5.71)	
*Escherichia coli*	1 (4.76)	0 (0.00)	0.375
*Streptococcus pneumoniae*	0 (0.00)	4 (11.43)	0.284
*Enterococcus faecalis*	2 (9.52)	1 (2.86)	0.646
Fungi, *n* (%)	5 (23.81)	4 (11.43)	0.398
*Candida albicans*	1 (4.76)	4 (11.43)	0.717
*Proximal smooth pseudomembranilli*	1 (4.76)	0 (0.00)	0.375
*Pseudomembranilli albicans*	3 (14.29)	0 (0.00)	0.092
No. of infecting pathogenic organisms, *n* (%)			
Combination of infection with one pathogenic organism	9 (42.86)	15 (42.86)	
Combination of infection with two pathogenic organisms	7 (33.33)	11 (31.43)	0.883
Combination of infection with three pathogenic organisms	3 (14.29)	3 (8.57)	0.823
Combination of more than three pathogenic bacteria infections	2 (9.52)	6 (17.14)	0.693

### 3.3 Pathogenic organisms and invasive procedures during ECMO maintenance

Table 5 shows that patients in the survivor group received ECMO and ventilator support for a longer period of time compared to the non-survivor group, with the duration of ECMO maintenance (95.50 h vs. 43.00 h, *p* = 0.011) and ventilator maintenance (132.50 h vs. 65.10 h, *p* = 0.034). The majority of patients in the non-surviving group developed infections as soon as ECMO was running (*p* < 0.05). However, there was no significant difference between the invasive procedure (e.g., CRRT, fibreoptic bronchoscopy, thoracentesis, tracheotomy, laparotomy, bone marrow aspiration, temporary pacemaker, etc.) that the two groups underwent during ECMO.

**Table 5:** Details and types of invasive procedures with causative organisms during ECMO maintenance.

Characteristics	Survivor group (*n* = 21)	Non-survivor group (*n* = 35)	*p*
ECMO maintenance time (hours), M (Q₁, Q₃)	108.00 (95.50, 160.00)	85.00 (43.00, 114.00)	0.011
Ventilator maintenance time (hours), M (Q₁, Q₃)	194.00 (132.50, 260.00)	90.30 (65.10, 237.50)	0.034
Stage of infection			
During ECMO operation	10 (47.62)	26 (74.29)	0.044
Within 48 hours of ECMO withdrawal	11 (52.38)	9 (25.71)	0.044
Invasive procedure during ECMO maintenance			
Continuous renal replacement therapy, *n* (%)			
Continuous veno-venous hemodialysis	9 (42.86)	11 (31.42)	0.48
Hemoperfusion	4 (19.05)	6 (17.17)	
Artificial liver	0 (0.00)	4 (12.12)	0.261
Fiberoptic bronchoscopy	14 (66.67)	29 (82.86)	0.288
Intra-aortic balloon pump	5 (23.81)	6 (17.17)	0.878
Thoracocentesis	5 (23.81)	7 (20.00)	
Tracheotomy	3 (14.29)	2 (5.71)	0.545
Abdominal puncture	2 (9.52)	2 (5.71)	
Bone marrow aspiration	2 (9.52)	0 (0.00)	0.136
Temporary pacemaker	1 (4.76)	2 (5.71)	
Other invasive maneuvers	4 (19.05)	3 (8.57)	0.465

### 3.4 Inflammatory markers at the time of first infection predict prognosis in patients with ECMO-associated infections

As shown in Table 6, IL-6, PCT, hs-CRP, WBC, NE, and Plt were not statistically significant in the surviving and non-surviving groups at ECMO initiation (*p* > 0.05). Table 7 shows that IL-6, hs-CRP and Plt at the time of first infection were significantly different between the surviving and non-surviving groups (*p* < 0.05), with IL-6 (40.62 vs. 196.75 μg/mL, *p* < 0.001), hs-CRP (8.86 vs. 23.60 mg/L, *p* < 0.001), Plt (85.00 vs. 48.50 × 10^9^, *p* = 0.02). PCT, WBC and NE were not significantly different between the two groups. Univariate logistic regression analysis in Table 8 showed that IL-6 (OR: 1.02, 95% CI: 1.01–1.03, *p* = 0.001), hs-CRP (OR: 1.59, 95% CI: 1.02–2.47, *p* = 0.041) and Plt (OR: 1.01, 95% CI: 1.01–1.02, *p* = 0.031) were significant predictors of prognosis in patients with ECMO-associated nosocomial infections.

**Table 6:** Inflammatory biomarkers at ECMO initiation.

Characteristics	Survivor group (*n* = 21)	Non-survivor group (*n* = 35)	*p*
IL-6 (ug/mL), M (Q₁, Q₃)	1.98 (1.80, 2.49)	1.77 (1.30, 2.28)	0.065
PCT (ug/L), M (Q₁, Q₃)	0.06 (0.04, 0.09)	0.04 (0.02, 0.05)	0.059
hs-CRP (mg/L), M (Q₁, Q₃)	4.04 (3.37, 5.61)	5.10 (3.89, 7.73)	0.255
WBC (×10^9^), M (Q₁, Q₃)	6.77 (4.50, 12.22)	8.90 (4.78, 13.11)	0.598
NE (*n* %), M (Q₁, Q₃)	46.75 (40.40, 62.06)	41.34 (30.45, 63.80)	0.555
Plt (×10^9^), M (Q₁, Q₃)	230.97 (168.73, 265.82)	214.04 (168.39, 240.89)	0.410

IL-6, interleukin 6; PCT, procalcitoninogen; hs-CRP, high-sensitivity C-reactive protein; WBC, white blood cell count; NE, neutrophil percentage; Plt, platelets.

**Table 7:** Trends in the transformation of inflammatory biomarkers at the time of first infection.

Characteristics	Survivor group (*n* = 21)	Non-survivor group (*n* = 35)	*p*
IL-6 (ug/mL), M (Q₁, Q₃)	80.45 (40.62, 108.02)	454.65 (196.75, 1089.17)	<0.001
PCT (ug/L), M (Q₁, Q₃)	10.61 (7.37, 16.11)	15.16 (11.16, 25.16)	0.19
hs-CRP (mg/L), M (Q₁, Q₃)	10.51 (8.86, 16.40)	39.16 (23.60, 69.71)	<0.001
WBC (×10^9^), M (Q₁, Q₃)	10.83 (9.35, 16.92)	11.73 (8.43, 16.81)	0.447
NE (*n* %), M (Q₁, Q₃)	82.10 (28.77, 86.50)	84.25 (78.95, 89.10)	0.270
Plt (×10^9^), M (Q₁, Q₃)	152.00 (85.00, 206.00)	88.50 (48.50, 122.75)	0.020

IL-6, interleukin 6; PCT, procalcitoninogen; hs-CRP, high-sensitivity C-reactive protein; WBC, white blood cell count; NE, neutrophil percentage; Plt, platelets.

**Table 8:** Univariate logistic regression analysis of inflammatory biomarkers at the time of first infection.

Characteristics	*β*	S.E.	*Z*	*p*	OR (95%CI)
IL-6 (ug/mL)	0.02	0.01	2.58	0.010	1.02 (1.01 ~ 1.03)
hs-CRP (mg/L)	0.46	0.23	2.04	0.041	1.59 (1.02 ~ 2.47)
Plt (×10^9^)	0.01	0	2.15	0.031	1.01 (1.01 ~ 1.02)

IL-6, interleukin 6; hs-CRP, high-sensitivity C-reactive protein; Plt, platelets.

## 4 Discussion

In this study, we describe the clinical epidemiology of nosocomial infections with ECMO. We focused on the association between the distribution characteristics of pathogenic bacteria, early inflammatory markers and the prognosis of patients with nosocomial infections in ECMO. The incidence of nosocomial infections in ECMO was 32.7%%, with a survival rate of 37.5% and a mortality rate of 62.5% after infection. This finding is generally consistent with previous studies (Selçuk et al., 2021). Finally, this study also found that the most common site of infection for ECMO-associated infections was the respiratory tract, and the main pathogen was *Acinetobacter baumannii*. Elevated interleukin 6 (IL-6) and reduced platelet counts at the time of first infection were shown to be associated with a higher risk of in-hospital mortality.

ECMO is used as a last resort to save critically ill patients and provide temporary support in cases of severe cardiopulmonary failure. However, ECMO application may also lead to a strong host inflammatory response (Wang et al., 2022), including immune system, coagulation cascade and endothelial dysfunction (Millar et al., 2016). The release of pro-inflammatory mediators increases vascular permeability, activates patient endothelial cells, and further promotes a systemic inflammatory response (Cui et al., 2022), which may increase the risk of ECMO-related nosocomial infections (Wang et al., 2022). Conversely, ECMO application may also reduce pro-inflammatory injury by improving organ perfusion and gas exchange (Rozencwajg et al., 2019). Therefore, early detection of inflammatory markers is important for disease management, prognostic assessment, and treatment selection (Liu et al., 2021; Drmić et al., 2023).

In our study, inflammation-related inflammatory markers including IL-6, hs-CRP, and Plt were found to correlate with the prognosis of ECMO nosocomial infections, which has been demonstrated in other infections (Selçuk et al., 2021; Wei et al., 2023; Stockmann et al., 2020). IL-6 is a polybiologically active cytokine that reflects inflammation in the body, which catalyzes and amplifies inflammatory responses, and is closely related to the severity of the disease (Wei et al., 2023). It has been found that when IL-6 exceeds 150 pg./mL, the mortality rate increases significantly (Smieszek et al., 2021); in this study IL-6 expression levels in the ECMO-associated nosocomial infections surviving and non-surviving groups were IL-6 (40.62, 108.02 vs. 196.75, 1089.17ug/mL, *p* < 0.001), respectively, and one-way logistic regression analysis IL-6 (OR:1.02, 95% CI: 1.01 ~ 1.03; *p* = 0.001) was obtained, and the results suggest that serum IL-6 levels may be useful in assessing disease progression.

Thrombocytopenia and poor prognosis have been demonstrated in several studies (Walker et al., 2024), and the risk of associated complications and short-term mortality may be significantly increased in patients with Plt reduction before receiving mobile ECMO support (Krasivskyi et al., 2023). Our study found that Plt was statistically significant at the first statistically significant time of ECMO-associated nosocomial infections in the survivor and non-survivor groups with data of (85.00, 206.00 vs. 48.50, 122.75 × 10^9^, *p* = 0.02), respectively, which is consistent with the previous relatively large decrease in Plt associated with an increase in in-hospital mortality in patients treated with VA-ECMO on day 1 (Wang et al., 2021), and that the one-way Factor logistic regression analysis further confirmed that early reduction in blood Plt in patients with ECMO-associated nosocomial infections may imply a poor prognosis. The reduction in Plt at this time may be linked to concomitant infections, disease-induced thrombosis, mechanical shear forces on Plt caused by the ECMO circuit, and systemic inflammatory responses. These factors may exacerbate Plt depletion or inhibit its generation through various pathways (e.g., the release of procoagulant factors). Hs-CRP has been studied as a predictor of clinical outcome in critically ill patients (Koozi et al., 2020). We found that in patients with ECMO-associated nosocomial infections, the first hs-CRP level was significantly different between the surviving and non-surviving groups, suggesting that it has some predictive value for prognosis. However, some studies have shown that early hs-CRP levels may not be a good predictor of survival in critically ill patients, while late hs-CRP may be more predictive (Koozi et al., 2020).

The study found that the risk of bacterial resistance during V-V ECMO therapy increased with the duration of ECMO support, particularly against gram-negative (GN) multidrug-resistant (MDR) bacteria (Boscolo et al., 2024). Specifically, the study showed that 21% of MDR GN bacteria were detected before ECMO connection, while 29% were identified after ECMO connection. Notably, the history of MDR GN bacteria isolated prior to ECMO connection was found to be an independent risk factor for mortality (Boscolo et al., 2024). Furthermore, the pharmacokinetic (PK) and pharmacodynamic (PD) effects of ECMO on antimicrobial drugs vary by drug class (Duceppe et al., 2021). For example, in the case of polymyxin B (PMB), it was observed that the difference in PMB concentration before and after the oxygenator was not significant, although the pharmacokinetics of PMB may be altered in patients treated with CRRT-ECMO (Xu et al., 2024). Similarly, ticlopidine (TEIC), used for secondary bacterial infections, did not show a significant effect on its pharmacokinetics during V-V ECMO therapy (Hirayu et al., 2022). Therefore, managing bacterial infections during ECMO therapy, especially preventing and controlling drug-resistant bacteria, is crucial. Additionally, the pharmacokinetics of antimicrobials must be tailored to the specific drug.

There are several risk factors contributing to hospital-acquired infections in patients undergoing ECMO-assisted therapy. Among these, the duration of ECMO support plays a crucial role in infection development. As the duration of ECMO therapy increases, the risk of infection rises significantly. A study found (Wang et al., 2022) that the incidence of infection was 9.5/1000 in patients receiving ECMO for 3 to 10 days, while it increased to 27.2/1000 and 64.5/1000 in those receiving ECMO for 11 to 20 days and 21 to 30 days, respectively. Longer ECMO support has been reported to further impair patients’ immune and coagulation functions, thereby increasing the incidence of hospital-acquired infections (Biffi et al., 2017). The development of infections may be attributed to the fundamental operation of ECMO, in which blood is exposed to the non-endothelialised surface of the ECMO circuit, leading to widespread activation of the innate immune system and triggering the production of various cytokines (e.g., tumor necrosis factor alpha, interleukin 6, interleukin 8) (Millar et al., 2016). Inflammatory markers such as CRP and PCT show a more pronounced inflammatory response in patients on mechanical circulatory support (MCS), particularly in critically ill ICU patients (Kreutz et al., 2024). Therefore, clinicians should conduct frequent assessments to shorten the duration of ECMO use and reduce the length of hospitalization, thereby mitigating the risk of infection.

In addition, previous hospitalization history is an important reference for assessing the outcome and prognosis of patients for re-hospitalization. In this study, we found that a history of invasive operations in the last month, antibiotic use in the last month, and glucocorticoid and immunosuppressant use in the last month were associated with ECMO hospital-associated infections, and similar results were confirmed in other studies (Han et al., 2019; Joshi et al., 2024). However, the relationship between ECMO-associated nosocomial infections and history of previous hospitalization requires further study.

## 5 Conclusion

The results of this retrospective study indicate that the most common site of infection during ECMO-assisted therapy is the respiratory tract and the most common pathogen is *Acinetobacter baumannii*. Elevated interleukin 6, elevated ultrasensitive C-reactive protein, and decreased platelet count are potential biomarkers for predicting the prognosis of patients with ECMO-associated nosocomial infections. As this study is a single-center retrospective study, the findings reflect local antibiotic resistance and pathogen patterns, which may limit their generalizability to other hospitals. Further multicenter studies are needed to develop effective interventions to reduce the mortality of ECMO-associated nosocomial infections.

## Data Availability

The original contributions presented in the study are included in the article/supplementary material, further inquiries can be directed to the corresponding author.
